# Diabetic Ketoacidosis among Diabetic Patients Admitted in the Department of Medicine of a Tertiary Care Centre: A Descriptive Cross-sectional Study

**DOI:** 10.31729/jnma.8158

**Published:** 2023-05-31

**Authors:** Lochan Karki, Milan Khadka, Milan Purna Oli, Santosh Joti, Rama Tamrakar, Siddhant Adhikari, Suman Khatri, Poonam K C

**Affiliations:** 1 Department of Medicine, National Academy of Medical Sciences, Mahaboudha, Kathmandu, Nepal; 2 Godawari Midcity Hospital, Satdobato, Lalitpur, Nepal; 3 Silverline Hospital, Balaju, Kathmandu, Nepal; 4 Lubhoo Primary Health Care Centre, Mahalaxmi, Lalitpur, Nepal; 5 Lumbini Provincial Hospital, Butwal, Rupandehi, Nepal; 6 Ganeshman Singh Memorial Hospital and Research Center, Mahalaxmisthan, Lalitpur, Nepal

**Keywords:** *diabetes mellitus*, *diabetic complications*, *diabetic ketoacidosis*, *Nepal*

## Abstract

**Introduction:**

Diabetic ketoacidosis is one of the most severe acute complications of diabetes mellitus characterised by hyperglycemia, hyperketonemia, and metabolic acidosis. Prompt diagnosis and treatment of diabetic ketoacidosis can decrease severity, hospital stay, and possible mortality. This study aimed to find out the prevalence of diabetic ketoacidosis among diabetic patients admitted to the department of medicine of a tertiary care centre.

**Methods:**

This descriptive cross-sectional study was conducted at a tertiary care centre. Data from 1 March 2022 to 1 December 2022 were collected between 1 January 2023 and 1 February 2023 from the hospital records. The ethical approval was taken from the Institutional Review Committee of the same institute (Reference number: 466/2079/80). All the diabetic patients admitted to the Department of Medicine during our study duration were enrolled for the study. Diabetic patients who left against medical advice and those with incomplete data were excluded from the study. Data were collected from the medical record section. Convenience sampling method was done. Point estimate and 95% Confidence Interval were calculated.

**Results:**

Among 200 diabetic patients, the prevalence of diabetic ketoacidosis was 7 (3.5%) (3.47-3.53, 95% Confidence Interval) among which 1 (14.29%) patients had type I diabetes mellitus and 6 (85.71%) had type II diabetes mellitus patients and the mean HbA1C level was 9.77%.

**Conclusions:**

The prevalence of diabetic ketoacidosis among diabetes mellitus patients admitted to the department of medicine of a tertiary care centre was found to be higher than in other studies done in similar settings.

## INTRODUCTION

Diabetes mellitus (DM) is a chronic metabolic condition of growing public health concern with an estimated prevalence of 8.5% in Nepal.^1,2^ Acute and long-term consequences of DM include diabetic ketoacidosis (DKA), hyperosmolar hyperglycemia, and hypoglycemia while receiving therapy. One of the most severe acute complications of DM is DKA.^3^ DKA is characterised by hyperglycemia, hyperketonemia and metabolic acidosis.^4^ DKA is associated with a high mortality rate if not treated.^5^

DKA if diagnosed in time, the patients can be prevented by an extensive hospital stay, increase in severity and possible mortality. As many of these cases are referred to and managed in a tertiary care centre, this research helps to give us an idea of DKA in such a centre.

This study aimed to find out the prevalence of DKA among diabetic patients admitted to the department of medicine of a tertiary care centre.

## METHODS

This descriptive cross-sectional study was conducted at the National Academy of Medical Sciences, Mahaboudha, Kathmandu, Nepal. Data from 1 March 2022 to 1 December 2022 were collected between 1 January 2023 and 1 February 2023 from the hospital records. The ethical approval was taken from the Institutional Review Committee of the National Academy of Medical Sciences (Reference number: 466/2079/80). All the diabetic patients admitted to the Department of Medicine with complete hospita data during our study duration were enrolled for the study. Diabetic patients who left against medical advice and those with incomplete data were excluded from the study. Data were collected from the medical record section. Convenience sampling technique was done. The sample size was calculated using the following formula:


$$\begin{array}{ll} \text{n} & ={\text{Z}}^{2}\times \frac{\text{p}\times \text{q}}{{\text{e}}^{\text{2}}}\\ & =1.96^{2}\times \frac{0.024\times 0.976}{0.03^{2}}\\ & =100 \end{array}$$

Where,

n = minimum required sample size
Z = 1.96 at 95% Confidence Interval (CI)
p = prevalence of DKA taken from previous studies, 2.4%^6^
q = 1-p
e = Margin of error, 3%

The calculated sample size was 100. After doubling the sample size, the sample size was 200.

Diabetes ketoacidosis is characterised by hyperketonemia, metabolic acidosis, and hyperglycemia. It is diagnosed as hyperglycemia ≥250 mg/dl, arterial blood pH ≤7.3, serum bicarbonate ≤18 mmol/l and the presence of ketonuria.^7^ Data were collected from the hospital record and records were categorised as type of diabetes (type I or type II), age groups, HbA1c level, smoking history, alcohol history, comorbidities and possible precipitating factors.

Data were entered in Microsoft Excel 2016 and analysis was done by using IBM Statistics SPSS 26.0. Point estimate and 95% CI were calculated.

## RESULTS

Among 200 Diabetic patients admitted to the Department of Medicine, 7 (3.5%) (3.47-3.53, 95% CI) had DKA with a mean HbA1c of 9.77±1.64% which ranges from 7.4% to 11.6%.

A demographic study of diabetic ketoacidosis patients showed that diabetic ketoacidosis was present among 1 (14.29%) male and 6 (85.71%) females and the highest number of patients with Diabetic ketoacidosis was found in the 45-59 years age group with the prevalence of 3 (42.85%) and with the mean age of 57±13.08 years. (Table 1).

**Table 1:** Demographic characteristics of the DKA patients (n= 7).

Gender	n (%)
Female	6 (85.71)
Male	1 (14.29)
**Age Group**	
30-44	1 (14.29)
45-59	3 (42.85)
60-74	2 (28.57)
>75	1 (14.29)

Majority of them, 6 (85.71%) had type II DM (Table 2).

**Table 2:** Type of diabetes among DKA patients (n= 7).

Types of DM	n (%)
Type I	1 (14.29)
Type II	6 (85.71)

Among possible precipitating factors for diabetic ketoacidosis to occur, infection was found to be the highest cause as all the patients were having infection (Table 3).

**Table 3:** Possible precipitating factors among diabetic ketoacidosis patients patients (n= 7).

Possible Precipitating factors	n (%)
**Infection**	7 (100)
Pneumonia	2 (28.57)
Dengue fever	1 (14.29)
Urinary tract infection	1 (14.29)
Others	2 (28.57)
**Acute Pancreatitis**	1 (14.29)

Among 7 diabetic ketoacidosis patients, there were nocomorbidities in 5 (71.43%) patients and hypertensionas a comorbidity was present in 1(14.29%) patient(Table 4).

**Table 4:** Comorbidities among DKA patients (n= 7).

Comorbidities	n (%)
Hypertension	1 (14.29)
Chronic Obstructive Pulmonary Disease	1 (14.29)
Chronic kidney disease	1 (14.29)
Dyslipidemia	1 (14.29)

## DISCUSSION

A descriptive cross-sectional study among 200 diabetes mellitus patients admitted in the department of the medicine of a tertiary care centre of Nepal was done which showed the prevalence of diabetic ketoacidosis was 3.5% which is higher than other similar studies.^6,8^ A study done in the Department of medical college of Tamil Nadu, India showed that the prevalence of diabetic ketoacidosis was 2.4%.^6^ Another study done in Benghazi Diabetes Centre, Libyan Arab Jamahiriya showed that among the hospital-admitted patient, diabetic ketoacidosis was present in 1.6% of the diabetic patients.^8^

In our study, among diabetic ketoacidosis patients, 85.71% were female and 14.29% were male with a mean age of 57±13.08 years. A study done in the tertiary care centre of Eastern Nepal showed that the mean age of diabetic ketoacidosis patients was found to be 48.2 years which is lower in comparison to our study.^9^ Another study on diabetic ketoacidosis patients done at University Teaching Hospital, Zambia had male to female ratio of 1 which is in contrast to our study, but the mean age was found to be 43.73±15.61 years which is lower in comparison to our study.^10^

The mean HbA1c level in our study showed 9.77±1.64 % ranging from 7.4% to 11.6%. A study done in the Dhulikhel Hospital of Nepal among diabetic ketoacidosis also showed that all the patients were having an HbA1c level above 7%.^11^ Also, the study in Tamil Nadu showed the mean HbA1c level among diabetic ketoacidosis patients was 12.1±2.7 %.^6^

Among the diabetic ketoacidosis patients, it was found that 14.29% were type I diabetic patients and 85.71% were type II diabetic patients in our study. In the study of Tamil Nadu as well, diabetic ketoacidosis was more among type II diabetic patients which consists of 67.6%.^6^ Studies done in the hospital of Eastern Nepal also showed a slight increase number of diabetic ketoacidosis patients with type II diabetes (9 patients) in comparison to type I diabetes (7 patients).^9^

In our study, it was found that infection was the most common possible precipitating factor for diabetic ketoacidosis with all the patients having the infection which is higher in comparison to other studies. In the study of Tamil Nadu, it was found that the infection was the most common precipitating factor among 58.1% of patients followed by non-adherence to the medication (20.9%).^6^ Another study done on the clinical profile and the treatment outcome of diabetic ketoacidosis showed that infection was the most common precipitating factor but only in 30% of cases.^12^ However, a study done in the emergency department of the tertiary care centre showed that 73.33% of patients with diabetic ketoacidosis had the infection as the precipitating factor.^13^

There were several limitations to our study. It was a pilot study with a small sample size from a medicine department of a single hospital in a short period. We did not have information on long-term outcomes because the study was retrospective. Also, as the hospital we studied is one of the major government hospitals of Nepal, most of the cases are referred from every corner of the country making our prevalence higher in comparison to other studies. Since we have done the descriptive cross-sectional study, we couldn't determine why diabetic ketoacidosis is more common among type II diabetes mellitus patients so further analytical studies need to be done.^8^

## CONCLUSIONS

The prevalence of diabetic ketoacidosis among diabetic patients admitted to the department of medicine in a tertiary care centre of Nepal is higher than other studies done in similar settings with higher prevalence among type II diabetes mellitus patients.
